# Insulin-Like Growth Factor-1 at Diagnosis and during Subsequent Years in Adolescents with Type 1 Diabetes

**DOI:** 10.1155/2018/8623560

**Published:** 2018-03-20

**Authors:** Simona I. Chisalita, J. Ludvigsson

**Affiliations:** ^1^Department of Endocrinology and Department of Medicine and Health Sciences, Linköping University, Linköping, Sweden; ^2^Division of Pediatrics, Department of Clinical and Experimental Medicine, Linköping University, Linköping, Sweden

## Abstract

**Background:**

Type 1 diabetes (T1D) in adolescents is associated with alterations in the insulin-like factor system probably caused both by a deranged metabolism and insulinopenia in the portal vein.

**Objective:**

To study how the circulating IGF-1 is affected at diagnosis and during subsequent years in adolescents with T1D.

**Methods:**

Ten girls and ten boys with type 1 diabetes (T1D), aged 13.0 ± 1.4 (mean ± SD) years at diagnosis, took part in the study. Blood samples were drawn at diagnosis and after 3, 9, 18, and 48 months. HbA1c, total IGF-1, and C-peptide were measured.

**Results:**

At diagnosis, the patients had high HbA1c, low IGF-1, and measurable C-peptide. After the start of insulin treatment, maximal improvement in glycemic control and IGF-1 occurred within 3 months and then both tended to deteriorate, that is, HbA1c to increase and IGF-1 to decrease. C-peptide decreased with time, and after 4 years, half of the patients were C-peptide negative. At diagnosis, C-peptide correlated positively to IGF-1 (*r* = 0.50; *p* < 0.03). C-peptide correlated negatively with insulin dose (U/kg) after 18 and 48 months from diagnosis (*r* = −0.48; *p* < 0.03 and *r* = −0.72; *p* < 0.001, resp.).

**Conclusions:**

In conclusion, our results show that in newly diagnosed adolescents with type 1 diabetes and deranged metabolism, the IGF-1 level is low and rapidly improves with insulin treatment but later tends to decrease concomitantly with declining endogenous insulin secretion.

## 1. Introduction

Insulin-like growth factor-1 (IGF-1), a peptide closely related to insulin, mediates the growth-promoting effects of growth hormone (GH) in children and the anabolic effects of GH in adults [1]. Circulating IGF-1 is mainly secreted by the liver under the control of GH. Insulin enhances the sensitivity of the liver to GH probably by upregulating GH receptors and thereby increasing IGF-1 production [2]. IGF-1 peaks during puberty and then levels off [3]. More than 99% of circulating IGF-1 is bound to the six IGF binding proteins (IGFBPs) of which the major binding protein IGFBP-3 acts as a natural store, prolonging IGF-1 half-life. IGFBPs modulate the biological activity of IGF-1 [4].

Type 1 diabetes (T1D) is associated with dysregulation of the GH–IGF system [5]. IGF-1 is lower in nondiabetic controls in spite of elevated GH [6–10]. These changes have been related to poor metabolic control and to lack of endogenous insulin production which causes insulinopenia in the portal vein and insufficient insulin delivered to the liver. A previous study has shown that treatment with intensified exogenous subcutaneous insulin therapy only attenuates these disturbances but does not correct them [11]. In T1D, intraperitoneal administration of exogenous insulin has a more pronounced effect on the dysregulated IGF system than that of subcutaneous insulin, that is, increasing circulating IGF-1 and decreasing GH and IGFBP-1 more than subcutaneous insulin therapy [12].

Our aim was to study how the dynamic changes in glycemic control and endogenous insulin secretion influence the total IGF-1 levels in adolescents at diagnosis of T1D and during subsequent years.

## 2. Materials (Subjects) and Methods

Ten girls and ten boys with T1D, aged 13.0 ± 1.4 (mean ± SD) years at diagnosis, took part in the study. Demographic data are given in Table 1.The period of puberty was in this study defined as 10.0–14.9 years for girls and 12.0–16.9 years for boys.

At diagnosis, nonfasting blood samples were drawn before treatment (0 months). Fasting blood samples were drawn after 3, 9, 18, and 48 months. Height and weight were measured at the same occasion. The patients received standard care—in this case, multiple daily injection insulin therapy based on active self-monitoring—and were seen at the outpatient clinic every 1–3 months. Insulin was administrated as mealtime injections of rapid acting insulin analogs, lispro, or aspart, in combination with basal insulin (NPH, detemir, or glargine) or as CSII with insulin pump.

### 2.1. Biochemical Analysis

HbA1c (reference range: 4.6–6.0% (NGSP), corresponding to 27–42 mmol/mol (IFCC)) was analyzed with reverse-phase partition chromatography on a cation exchanger column using high-performance liquid chromatography (HPLC; Auto A1C HA 8110, Boehringer Mannheim). Analyses of C-peptide were done with time-resolved fluoroimmunoassay (AutoDELFIA TM, C-peptide kit, Wallac, Turku, Finland) with a detection level of 0.03 nmol/l. Validation of each assay was performed using a C-peptide control module with a low-, medium-, and high-level control (Immulite, DPC, UK). MultiCalc programme (Wallac) was used for calculation of C-peptide levels. Total serum IGF-1 was measured by a one-step enzyme-linked immunosorbent assay (ELISA) after acid-ethanol extraction from its binding proteins with a commercial kit from Diagnostic System Laboratories (Webster, Texas, USA). The assay was performed according to the manufacturer's protocol.

### 2.2. Ethics

The study was conducted in accordance with the World Medical Association Declaration of Helsinki regarding ethical conduct of research involving human subjects and was approved by the Research Ethics Committee, Linköping University, Sweden.

### 2.3. Statistical Analysis

Statistical comparisons were made using SPSS 21.0 for Windows software (IBM Statistics, New York, USA). Mean and standard deviation or medians and interquartile range are reported for continuous variables, and number and percentage are reported for categorical variables. Comparisons between groups were done using Student's *t*-test or ANOVA if three or more groups were compared. Correlations were done using Pearson correlation coefficient (*r*) or if nonlinear association Spearman's correlation coefficient (rho). All statistical tests were performed at the 5% significance level.

## 3. Results

### 3.1. Metabolic Control

**Figure 1:**
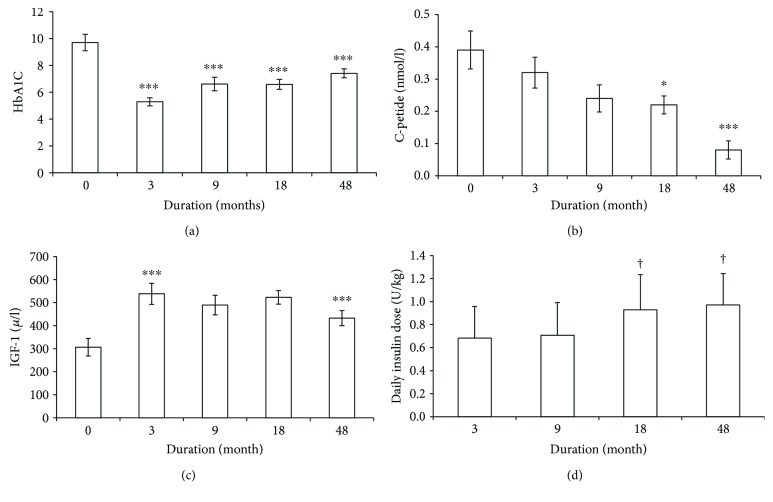
(a) Glycaemic control by HbA1C (%); (b) C-peptide (nmol/l); (c) IGF-1 (*μ*g/l); and (d) insulin dose (U/kg) at diagnosis and 3, 9, 18, and 48 months after diagnosis and treatment with multiple insulin injection regimes (mean ± SD). ^∗^ *p* < 0.05 and ^∗∗∗^ *p* ≤ 0.001 in comparison to diabetes onset; ^†^ *p* < 0.05 in comparison to 3 months.

Initially, all 20 patients (Table 1) had very high HbA1c which was markedly improved (6.2 ± 1.3% NGSP/45 ± 14 IFCC, *p* < 0.001) after treatment for 3 months with multiple injection insulin therapy (MIT) (Figure 1(a)). Good glycemic control was maintained at 9 months and 18 months but had deteriorated 48 months after diagnosis (8.3 ± 1.4%, *p* < 0.011 compared to 3 months) (Figure 1). Boys tended to have lower HbA1c values at diagnosis and a better glycemic control during the follow-up period than girls (*p* = 0.06 at 9 months, Supplementary Figure 1). During the follow-up period of 48 months both boys and girls continued to grow and the length attained after 48 months was 166 ± 5 cm for girls and 183 ± 8 cm for boys.

### 3.2. C-Peptide

At diagnosis all patients had measurable C-peptide 0.3 (0.1–0.9) nmol/l (median, IQ range) in random nonfasting blood samples. Fasting C-peptide gradually decreased from 3 months after diagnosis (Figure 1(b)). After 48 months, less than half of the patients had detectable C-peptide and there was a sex difference with lower endogenous insulin secretion in boys (*p* < 0.03).

### 3.3. IGF-1

At diagnosis, the patients had low total IGF-1, 307 ± 167 *μ*g/l, which increased markedly to 538 ± 198 *μ*g/l (*p* = 0.001) 3 months after diagnosis (Figure 1(c)). High values of IGF-1 were still found at 9 and 18 months after diagnosis (499 ± 190 *μ*g/l, *p* = 0.04 and 523 ± 30 *μ*g/l, *p* = 0.001, resp.), but then IGF-1 declined and after 48 months, IGF-1 was no longer significantly different from the value at diagnosis. The IGF-1 levels were similar in boys and girls.

### 3.4. Insulin Treatment

At 3 months' duration, the average insulin dose was 0.7 ± 0.3 U/kg and the insulin dose increased with increasing duration being 1.0 ± 0.3 U/kg at 48 months (*p* = 0.021) (Figure 1(d)). The insulin dose increased more in boys than in girls (*p* = 0.02) at 48 months (Supplementary Figure 2).

### 3.5. Correlations

**Figure 2:**
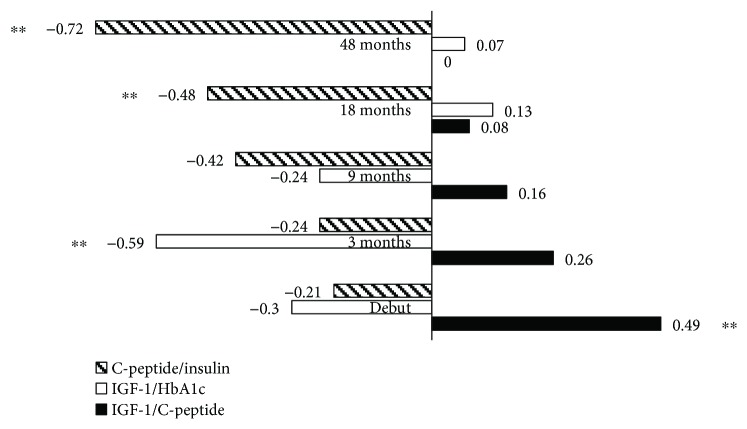
Pearson correlation of C-peptide to daily insulin doses/kg, IGF-1 to HbA1c, and IGF-1 to C-peptide levels. ^∗∗^ *p* < 0.01.

At diagnosis, there was a positive correlation between C-peptide and IGF-1 (*r* = 0.50; *p* < 0.03). There was a negative correlation between C-peptide and insulin dose at 18 and 48 months after diagnosis (*r* = −0.50; *p* < 0.031 and *r* = −0.72; *p* < 0.001, resp.) (Figure 2).

HbA1c values were negatively correlated to IGF-1 (*r* = −0.6, *p* < 0.008) after 3 months of insulin treatment (Figure 2).

No significant correlation was shown between insulin requirement and IGF-1 under follow-up period (data not shown).

## 4. Discussion

Low IGF-1 in type 1 diabetes as found in this study at diabetes diagnosis in adolescents has been ascribed to insulin deficiency as well as poor glycemic control [5, 8]. The positive correlation between endogenous insulin secretion measured by C-peptide and circulating IGF-1 at diagnosis is in line with the concept that the endogenous insulin secretion stimulates the IGF-1 production. Treatment with exogenous insulin and improvement of metabolic control increased circulating IGF-1 markedly within 3 months in agreement with previous observations [6, 13]. During insulin treatment, we found no association between C-peptide and IGF-1 in agreement with the observation of Sorensen et al. in adolescents [14]. In another study, Kim and Lee [15] found that in insulin-treated diabetic adolescents with good glycemic control, there was no association between C-peptide and IGF-1 levels while an association was found in adolescents with poor glycemic control. Bizzarri et al. [16] found IGF-1 to be related to C-peptide, HbA1c, puberty, and female gender. The role of C-peptide or endogenous insulin secretion on IGF-1 levels during insulin treatment is therefore not settled.

In our study, fasting C-peptide decreased with increasing diabetes duration and was very low after 4 years in agreement with a previous report [17]. The insulin dose expressed as U/kg/24 hrs increased with increasing duration, and there was a negative correlation between C-peptide levels and insulin doses. At 48 months diabetes duration, there tended to be a sex difference with lower endogenous insulin secretion and higher insulin doses in boys similar to what has been reported by Bizzari et al [16].

HbA1c decreased rapidly when insulin treatment was initiated at diagnosis and reached a nadir after 3 months when HbA1c was normal or near normal in most adolescents. However, thereafter, HbA1c increased. Probably, this worsening of glycemic control is at least partly due to the decline in the endogenous insulin secretion as preserved endogenous insulin secretion promotes good glycemic control [18–20], and we noticed a negative correlation between C-peptide and HbA1c at 18 months (*r* = 0.40; *p* = 0.09). As mentioned above, IGF-1 increased markedly when insulin treatment was started and had almost doubled after 3 months but did not increase further during the observation period of 48 months. The time course for the effect of insulin treatment on IGF-1 levels has been studied in detail in previous reports [6, 13]. A rise in IGF-1 was seen already after 1 day, and after 1 month, the IGF-1 level did not differ significantly from nondiabetic controls [21]. It may therefore be assumed that the effect of insulin treatment on the IGF-1 level had reached a steady state after three months. The IGF-1 level thereafter tended to decrease, and at 48 months, the difference from diabetes diagnosis was no longer significant. The reason for the decline in IGF-1 may be attributed either to a decline in GH secretion, having reached its maximum during puberty, or to a decline of endogenous insulin action, as the exogenously administrated insulin dose was not lowered at 48 months, but there was a decrease in endogenous insulin secretion. There is evidence that the IGF-1 production is especially dependent on insulin delivery through the portal vein which is why the reduced endogenous insulin secretion probably is of importance for the decline in the IGF-1 level [10, 12]. GH-secretion reaches its peak during puberty accompanied by a peak in the IGF-1 level at about 15 years of age [22]. A similar IGF-1 peak is also seen in adolescent with type 1 diabetes, but the peak is lower and may be delayed in girls [23]. Furthermore, Hedman et al. has been shown that in T1D, there is a decrease in IGF-1 concentrations together with low concentrations of IGFBP3 and high concentrations of IGFBP-1 [10]. The age range of our children at the start of the study was 11–15 years, and after 4 years, 15–19 years thus encompassing the age for the IGF-1 peak. It is conceivable that both the decline in endogenous insulin secretion and a decrease in GH secretion with age contributed to the decrease in the IGF-1 level after 48 months [4].

Poor glycemic control as observed at diabetes onset in this study is associated with low IGF-1 [6, 13]. After 3 months, we found a negative correlation between HbA1c and IGF-1 but not at other time points. A negative association between HbA1c and IGF-1 has been reported in some studies [14, 24] but not in others [15]. In adult type 1 diabetic patients with fair glycemic control, IGF-1 is not associated with HbA1c [25] while the presence of endogenous insulin secretion influences IGF-1 level independently of glycemic control [10]. Shiva et al. [13] concluded that the reduction of IGF-1 in children with type 1 diabetes is caused by the lack of insulin action and reversed by insulin therapy, a conclusion which also fits to some extent with our data, although our data supports the special importance of endogenous insulin secretion.

Dysregulation in the IGF-1 system in type 1 diabetes is associated not only with pure metabolic control but also with the development of severe complications such as end-stage renal disease and diabetes enteropathy [26, 27].

We acknowledge the limitation by the low number of included children with type 1 diabetes. In our study, we analyzed the total IGF-1 and we did not had the possibility to analyzed the bioactive IGF-1 which has been shown to be relatively more suppressed than total IGF-1 in children and adolescents with T1D [18].

In conclusion, our results show that in newly diagnosed adolescents with type 1 diabetes and deranged metabolism, IGF-1 levels are low and rapidly improving with insulin treatment but later tend to decrease concomitantly with declining endogenous insulin secretion.

## Figures and Tables

**Table 1:** Subject characteristics at diabetes diagnosis.

	All	Girls	Boys	*p* value
	(*n* = 20)	(*n* = 10)	(*n* = 10)	
Age (years)	13.0 ± 1.4	13.2 ± 1.8	12.7 ± 0.9	*p* = 0.45
Length (cm)	161 ± 11	160 ± 10	162 ± 14	*p* = 0.82
Weight (kg)	45 ± 19	43 ± 22	48 ± 16	*p* = 0.64
HbA1c (NGSP) (%)	10.5 ± 2.5	11.5 ± 3.1	9.6 ± 1.6	*p* = 0.15
HbA1c (IFCC) (mmol/mol)	91 ± 27	102 ± 34	82 ± 17	*p* = 0.15
C-peptide (nmol/l)	0.3 (0.1–0.9)	0.3 (0.1–0.9)	0.3 (0.1–0.7)	*P* = 0.48
IGF-1 (*μ*g/l)	307 ± 167	336 ± 170	281 ± 169	*p* = 0.49

## Abbreviations

T1D: Type 1 diabetes
IGF-1: Insulin-like growth factor-1
GH-IGF: Growth hormone-insulin-like growth factor axis.

## Supplementary Materials

Supplementary 1 Figure 1: HbA1c levels in boys and girls during follow up. At 9 months, the difference tended to be significant (*p* = 0.057).

Supplementary 2 Figure 2: daily insulin dose (U/kg) in girls and boys. The insulin dose tended to be higher in boys than in girls (*p* = 0.058) at 48 months.
