# Supplementary material for: Insulin-Like Growth Factor-1 at Diagnosis and during Subsequent Years in Adolescents with Type 1 Diabetes
Source: J Diabetes Res. 2018 Mar 20;2018:8623560. doi: 10.1155/2018/8623560 (PMC5883934; doi:10.1155/2018/8623560)

Supplementary Figure 1.

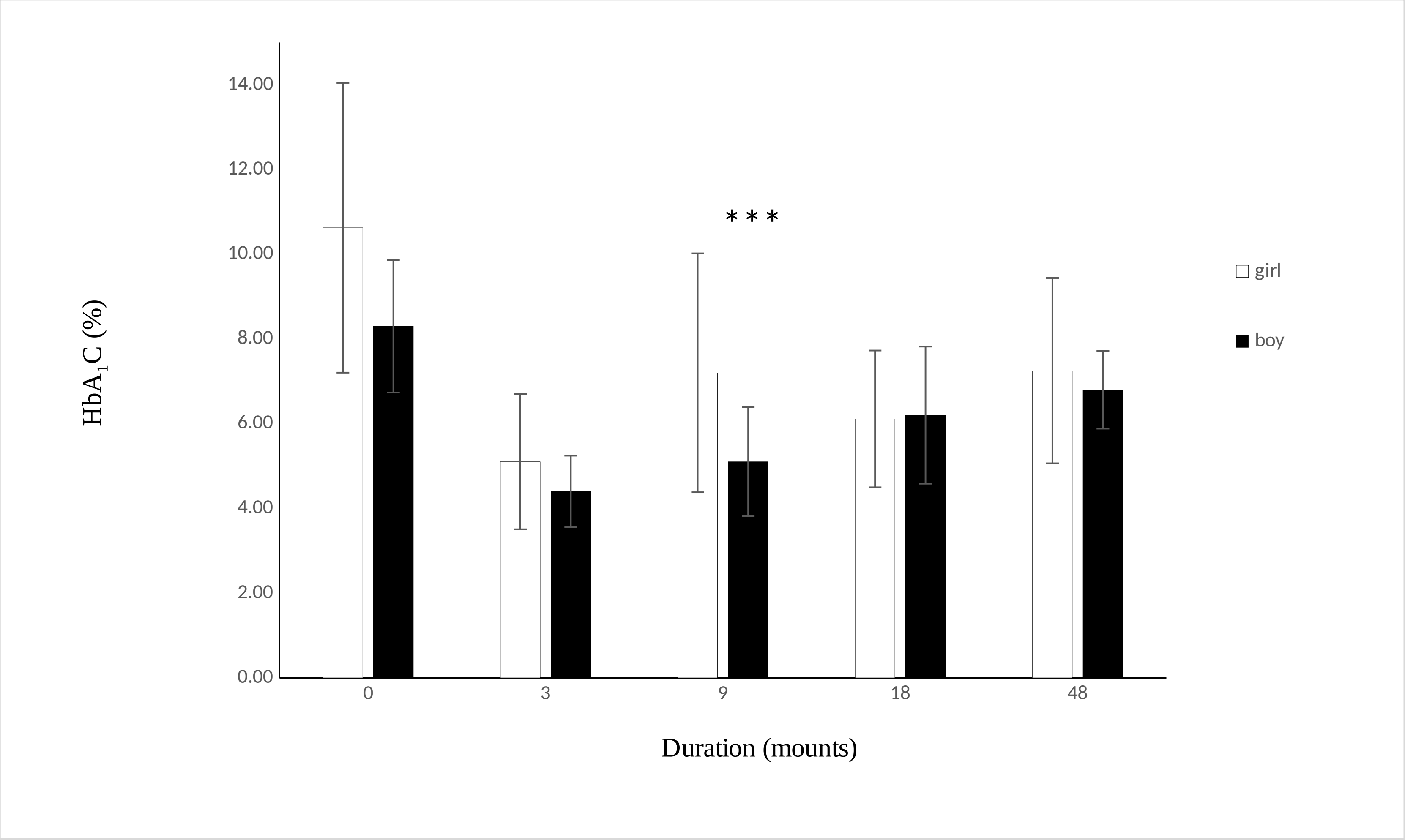

Supplement: Supplementary file 1 [file 8623560.f1.docx]
